# *Mycobacterium uberis* Infection in the Subcutaneous Tissue of the Radius/Ulna Area of a Cow

**DOI:** 10.3390/microorganisms8111701

**Published:** 2020-10-30

**Authors:** Lorraine Michelet, Maria Laura Boschiroli

**Affiliations:** Laboratory for Animal Health, Tuberculosis National Reference Laboratory, ANSES, University Paris-Est, 94700 Maisons-Alfort, France; maria-laura.boschiroli@anses.fr

**Keywords:** *Mycobacterium uberis*, *M. leprae*-like mycobacterium, radius/ulna area, cattle, bovine tuberculosis, diagnosis, interference

## Abstract

*Mycobacterium uberis* (*M. uberis*) is a recently described non-tuberculous mycobacterium phylogenetically close to *Mycobacterium leprae* (*M. leprae*) and *Mycobacterium lepromatosis* (*M. lepromatosis*). This pathogen classically causes nodular thelitis in cattle and goats. Here, we discuss what seems to be the first described case of *M. uberis* infection in a novel anatomical site, in the proximal or distal position (information not available) of the radius/ulna area of a cow. As this case was discovered in the framework of bovine tuberculosis (bTB) surveillance program in France, this type of infection could interfere with the screening and diagnostic tools employed for bTB.

## 1. Introduction

The genus *Mycobacterium* is a large genus that encompasses over 200 officially described species [1]. Except for the *Mycobacterium tuberculosis* complex (MTBC) and *M. leprae*, which are intracellular bacteria, mycobacteria are considered saprophytic and commonly found in the environment such as soil, water, and sediments [2]. Non-tuberculous mycobacteria (NTM) pose two main problems for animal and human public health: they are potentially responsible of opportunistic infections for humans and animals leading to significant economic losses and they can interfere in the diagnosis of bovine tuberculosis and other major mycobacterioses [3]. 

*Mycobacterium uberis* is a recently described mycobacterium, genetically close to the leprosy-causing bacilli *Mycobacterium leprae* and *Mycobacterium lepromatosis* [4]. Leprosy, due to *M. leprae* and *M. lepromatosis*, was considered as a disease of humans; however, it has been described in several animal hosts, including armadillos, red squirrels and non-human primates [5]. Eurasian red squirrels are currently the only known wild rodents carrying the leprosy bacilli and are considered a reservoir for leprosy in the British Isles [6,7]. *M. uberis* has been described as the causative agent of nodular thelitis in dairy animals [8,9]. Nodular thelitis due to this mycobacterium was originally described in France affecting cows [10,11]. The clinical manifestation of this pathology is the occurrence of chronic skin teat lesion with three stages: (1) localized inflammation, (2) oedema and spreading inflammation and (3) ulceration [11]. The use of next generation sequencing of the mycobacterium’s genomic DNA extracted directly from animal lesions made possible the description of this non-cultivable pathogen. Indeed, all attempts to grow the agent responsible for nodular thelitis in artificial media have failed [8,9,11]—a common trait shared with *M. leprae* due to their highly reduced genomes [4].

Bovine tuberculosis (bTB) caused by *Mycobacterium bovis* is a transmissible notifiable disease whose prevalence in cattle herds in Europe has been increasing in spite of long lasting and costly control campaigns [12,13,14]. France was declared officially bTB free in 2001 by the EU; however, *M. bovis* infection is frequent in cattle and wildlife, in particular in the Nouvelle-Aquitaine region [15]. The surveillance system in France is based on the ante mortem screening (single intradermal cervical test (SICT), single intradermal cervical comparative test (SICCT) and the Interferon gamma test (IFN-γ)) in cattle [16]. Diagnosis of bTB, as recommended by the EU, is based on bacteriology, histology and in France also by PCR. The case presented was discovered in the framework of the annual bTB screening test.

## 2. Case Report

A four year old “Prim’Holstein” cow was slaughtered in March 2019, as part of the bTB surveillance program in France, after a suspected positive result with the stringent interpretation of SICCT (Δ PPDb − Δ PPDa = 1.2 mm and Δ PPDb ≥ 2.4 mm). This cow belonged to a dairy herd, from the Pyrénées-Atlantiques department, in South-west France (Nouvelle-Aquitaine region). 

Veterinary inspection at the slaughterhouse revealed a subcutaneous lesion at the left anterior radius/ulna area, suggesting actinomycose or tuberculosis. Samples of the lesion as well as lymph nodes targeted for bTB diagnosis (retropharyngeal, tracheobronchial, mediastinal and mesenteric) were first submitted to first-line bTB diagnosis (bacteriology/PCR and histopathology) by authorised regional laboratories (RL) of the bTB national surveillance network [17]. Briefly, histopathology was based on Hematoxylin-Eosine and Ziehl Neelsen staining. DNA extraction was performed after mechanical lysis using an LSI MagVetTM Universal Isolation Kit (Life Technologies) with a KingFisherTM Flex automate (Thermo Scientific), following the manufacturer’s instructions. The LSI VetMAX^TM^ MTBC Real-Time PCR kit (Life Technologies), which targets IS*6110*, is used for MTBC detection. Bacterial culture was performed following the protocol established by the French national reference laboratory (NF U 47–104) for isolation of *M. bovis*. Amounts of 2 to 5 g of sampled tissues were crushed with a 4% sulfuric acid solution to decontaminate the tissue. After 10 min, the acid was neutralised by adding a 6% sodium hydroxide solution. After decontamination, the supernatant was seeded on two different media: Löwenstein–Jensen and Coletsos. All seeded media were incubated at 37 °C +/− 3 °C for three months and examined every 2 weeks. 

Histological changes consistent with mycobacteriosis were present in the subcutaneous lesion. There were large foci of necrosis surrounded by a granulomatous inflammation with multinucleated giant cells of the Langhans type. No acid fast bacilli were identified within lesions after Ziehl–Neelsen staining. DNAs extracted from the different samples (LN and cutaneous lesion) were all negative with MTBC PCR (Table 1). In case of positive histology and negative first line PCR—as for the cutaneous lesion—the DNA is sent to the national reference laboratory (NRL) for further characterization [18]. Identification of non-tuberculous mycobacteria species was performed at the NRL by sequencing the 65 kDa heat shock protein gene (*hsp65*) [19]. The obtained sequences were compared to the GenBank/EMBL/DDBJ databases using the BLAST program. They showed 100% identity with previously deposited *M. uberis* sequences (Genbank KJ095005 and KT599102) [8,9]. *M. uberis* was also confirmed using specific primers [4]. Bacteriological culture of this sample remained negative after three months. 

## 3. Discussion

This is the first description of *M. uberis* in the subcutaneous tissue of the radius/ulna area of a cow. Previously described sites of infection were mainly the udder or teat [8,9], but other locations—i.e., on the scrotum or on the flank—had also been described in goats [20]. To our knowledge, no other animal of this cattle herd showed cutaneous lesions or nodular thelitis. Previous studies suggest that milking operations may be implicated in infection transmission and that a greater occurrence of the disease is observed in 4–8 year-old cows or goats showing optimum milk production [10,20]. A wide range of clinical manifestations in humans and animals are caused by cutaneous mycobacterial infections, such as Buruli ulcer caused by *M. ulcerans* and other related slow growing mycobacteria, leprosy caused by *M. leprae* and *M. lepromatosis,* cutaneous manifestations of *M. tuberculosis* infection, and cutaneous infections caused by rapidly growing mycobacteria [21,22]. 

This is also the first description of *M. uberis* in the South of France (Table 2). Goat cases have been described in North-Western France [8,20]. Bovine cases have been described in the Jura and the Rhône departments, Eastern France [9,11]. The Pyrénées-Atlantiques region is currently among the most prevalent departments for bovine tuberculosis in cattle and in wildlife species (badgers and wild boar) in France [15]. This department gathers a third of the annual outbreaks. Screening tests are carried out on cattle every year in municipalities where at least one farm has been detected as an outbreak. As described in this article, *M. uberis* may interfere in the surveillance program of bTB, as non-specific reactions to the skin test, which have already been observed before [23], may occur. Additionally, the histological pattern observed in the cow lesion was compatible with tuberculosis. This case highlights the need of discriminating diagnostic tools such as molecular diagnosis that are demonstrated here to be a powerful method for differentiating non-tuberculous mycobacterioses from TB [18] to exclude any bTB suspicion. Bacteriology is not adapted for this bacterium due to its highly reduced genome. Indeed, attempts to cultivate mycobacteria from cows with nodular thelitis were unsuccessful [9].

## Figures and Tables

**Table 1:** Summary of bovine tuberculosis (bTB) surveillance tests results.

Sample	Bacteriology	Histopathology	MTBC PCR	Molecular Identification
Retropharyngeal LN ^1^	Negative	Negative	Negative	NA ^2^
Mediastinal LN ^1^	Negative	Negative	Negative	NA ^2^
Tracheobonchial LN ^1^	Negative	Negative	Negative	NA ^2^
Mesenteric LN ^1^	Negative	Negative	Negative	NA ^2^
Subcutaneous tissue	Negative	Positive	Negative	*M. uberis*

^1^ LN: Lymph node, ^2^ NA: not applicable.

**Table 2:** Comparison of the *M. uberis* cases identified in France.

	Study 1	Study 2	Study 3
Year of discovery	2013	2012–2013	2019
French Region	Jura (North-east)	Pays de la Loire (Center)	Nouvelle Aquitaine (South-west)
Host	Cow	Goat	Cow
Type of herd	Dairy	Dairy	Dairy
Size of the herd	30	600	144
Number of cases with lesion	10	100	1
Lesion Location	Teat	Udder/teat	Radius/ulna area
Bacteriology	Negative	Negative	Negative
Histology	Granulomatous dermatitis, necrosis	Severe granulomatous dermatitis, necrosis	Granulomatous inflammation, necrosis
Ziehl Neelsen staining	Positive	Negative	Negative
Diagnosis	Nodular thelitis	Nodular thelitis	Subcutaneous mycobacteriosis
Molecular identification	*M. uberis*	*M. uberis*	*M. uberis*
Reference	Pin et al., 2014 [9]	Chartier et al., 2016 [8]	This study
